# Improvements in Social Quality of Life for Recovering Addicts Receiving Dental Implant Rehabilitation

**DOI:** 10.7759/cureus.110049

**Published:** 2026-06-01

**Authors:** Austin F Crossanoff, William L Balanoff

**Affiliations:** 1 Curriculum and Academic Development, Institute for Dental Implantology, Jacksonville, USA; 2 Research and Development, Institute for Dental Implantology, Jacksonville, USA

**Keywords:** addiction recovery, depression prevention, implant-retained dentures, inflammation, mental health, patient health questionnaire (phq-9), periodontitis, social determinants of health (sdoh), substance use disorder (sud), systemic health

## Abstract

Substance use disorders (SUDs) are associated with severe oral deterioration, chronic systemic inflammation, and elevated depression risk, yet definitive dental rehabilitation is rarely integrated into long-term recovery care. This IRB-approved retrospective cohort study evaluated the effect of full-arch implant rehabilitation on depressive symptoms in 101 consecutively treated adults with verified long-term recovery from SUDs. Patients completed a modified PHQ-9 at baseline and again at final prosthesis delivery. Mean scores decreased from 24.16 preoperatively to 0.75 postoperatively, an 86.7% improvement, and all 101 patients reported better scores. Preoperatively, 80% (N=81) met the severe depression threshold (≥20); postoperatively, 92% (N=93) fell within the minimal-to-no depression range (0-4). These gains far exceed conventional thresholds for clinically meaningful change and are consistent with evidence linking resolution of oral inflammation to reduced systemic inflammatory burden and depression. These findings support full-arch implant rehabilitation in stabilized recovery populations as a scalable, high-value biopsychosocial component of comprehensive SUD care, rather than an elective procedure.

## Introduction

Substance use disorders (SUDs) give rise to widespread and accelerating consequences across the American healthcare landscape, shaping both individual outcomes and population-level mortality trends. Drug overdose is now the leading cause of unintentional injury death in the United States among young and middle-aged adults [1,2]. Synthetic opioids such as fentanyl have driven the ongoing epidemic and inflicted profound biological, psychological, and socioeconomic harm on individuals and communities globally. Individuals with SUDs experience all-cause mortality approximately three to four times higher than the general population, and they carry a disproportionate burden of cardiovascular, hepatic, infectious, and psychiatric comorbidities [3,4]. Within this broader pattern of systemic vulnerability, the oral health consequences of chronic opioid exposure represent a particularly overlooked domain of morbidity that warrants focused attention.

Among the least addressed but most debilitating consequences of addiction are oral diseases. Chronic exposure to acidic substances like opioids alters salivary pH, causes malnutrition, and leads to neglect of hygiene, which altogether cause rampant periodontitis and edentulism [5]. Periodontitis, together with dental caries, represents one of the most prevalent oral health threats worldwide, affecting nearly half of U.S. adults aged 30 and over and about 70% of those aged 65 and over [6,7]. The persistent bacterial inflammation in and beyond the oral cavity caused by chronic periodontitis leads to progressive destruction of the periodontal ligament and alveolar bone [8], oftentimes requiring elective surgeries to correct the deterioration. Individuals with opioid use disorder exhibit systemic immune dysregulation characterized by abnormal elevations in pro-inflammatory cytokines [9,10]. Additionally, reduced or altered neutrophil function further contributes to heightened infection risk and chronic inflammation [11-13]. Chronic opioid exposure is associated with xerostomia (dry mouth) and a significantly more acidic salivary pH [5], and this combination increases plaque accumulation and calculus buildup, which increases the likelihood of caries and periodontal disease [14]. The clinical consequences of oral deterioration extend beyond physiological damage, setting the stage for a cascade of psychosocial challenges that compound the burden of addiction.

Beyond the functional and mechanical impairments, tooth loss resulting from chronic periodontitis imposes significant psychosocial burdens that further hinder recovery. Evidence consistently links edentulism with heightened depressive symptoms, diminished self-esteem, and reduced overall quality of life, while dental implant rehabilitation has been shown to improve many of these effects by restoring appearance, mastication, and social confidence [15,16]. For individuals in sustained recovery, concerns about facial aesthetics, speech, and oral function frequently emerge as barriers to employment, stable relationships, and community reintegration. As a result, timely and comprehensive oral rehabilitation in appropriately stabilized patients serves not only as a clinical intervention, but as a critical component of long-term recovery support, enhancing psychological well-being and facilitating socioeconomic re-entry.

## Materials and methods

**Figure 1:**
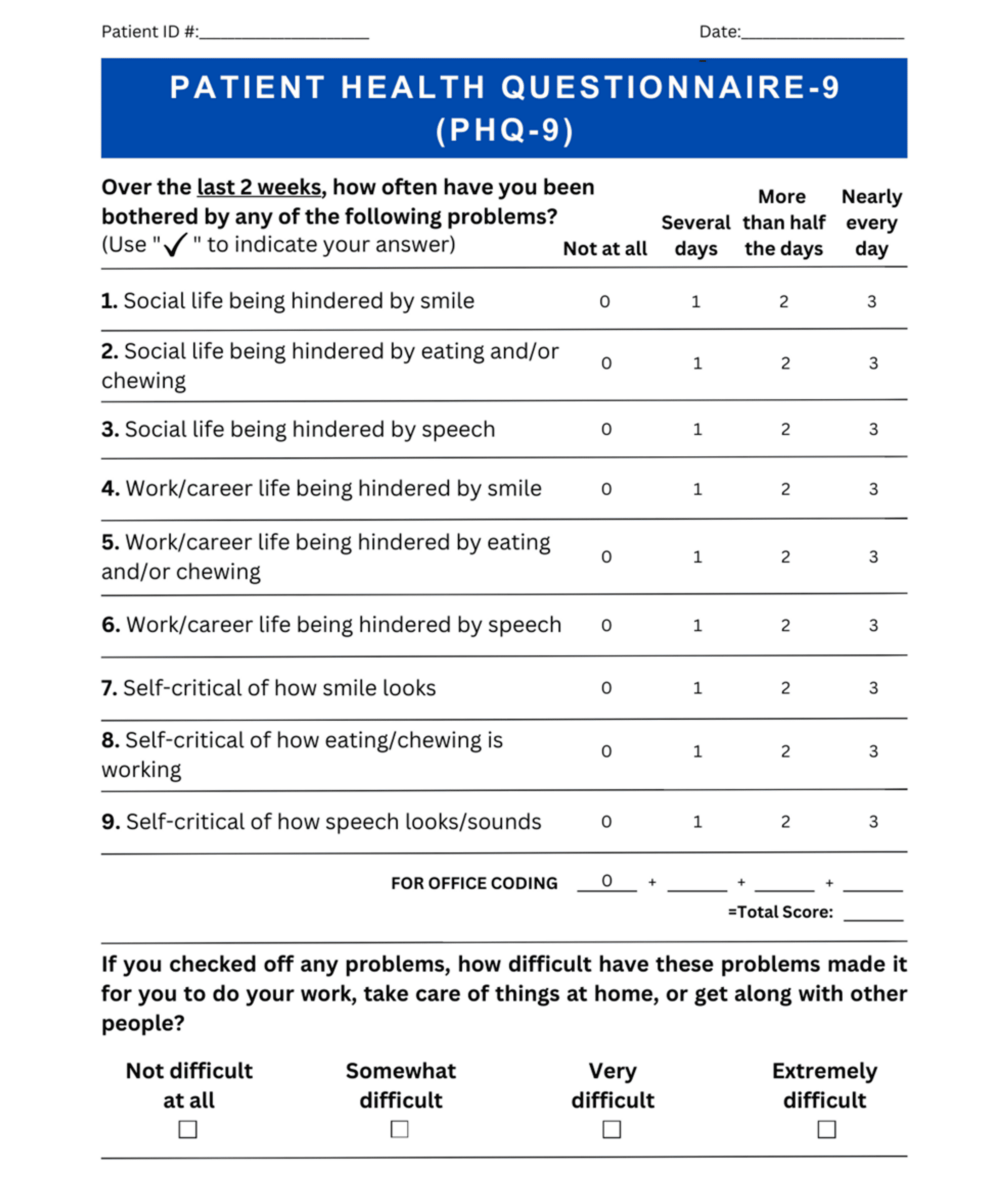
Patient Health Questionnaire-9 (PHQ-9)

Sterling IRB issued exemption from IRB review (IRB ID 14431-WBalanoff) in the Human Subjects under the Ethics Statement and Conflict of Interest Disclosures for this study. All patients completed the necessary informed consent and release-of-records documentation, permitting the use of their de-identified medical and dental data in research analyses. Participants were enrolled consecutively over a six-month period (June 2025 - December 2025) as post-op PHQ-9 questionnaires were integrated into the surgical protocol. During the initial comprehensive examination, each patient completed a baseline PHQ-9 assessment, which is an in-house document developed to incorporate questions related to social determinants of health (SDoH) and patient satisfaction with their oral condition prior to treatment (Figure 1). The same questionnaire was administered a second time at the end of the surgical process once healing was complete and patients returned for their final fitting appointment (typically 4-6 months after surgery). Eligible patients were at least 18 years of age, received or were planning to receive surgery after January 1st, 2025, and completed final recovery and post-op PHQ-9 questionnaires during the six-month study period window.

**Figure 2:**
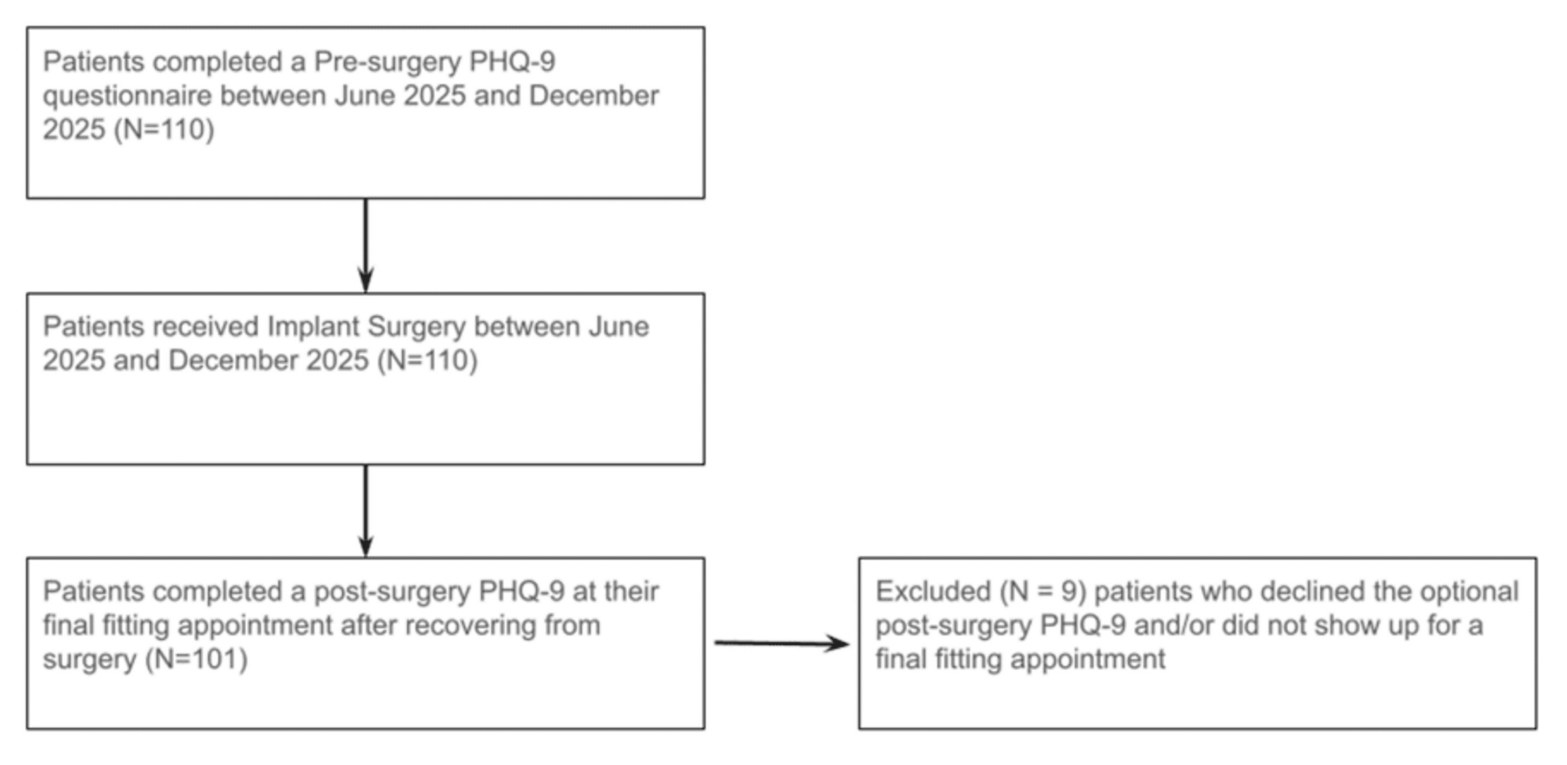
Flowchart of Cohort Selection PHQ-9: Patient Health Questionnaire-9

After applying these criteria, an initial study population of n=110 patients was identified. Several individuals lacked a postoperative PHQ-9 in their records and were contacted by telephone to complete the assessment with trained medical or dental staff. Patients who declined participation in the PHQ-9 surveys were excluded, resulting in a final sample of n=101 patients (see Figure 2 for a complete cohort selection flowchart). Medical records were reviewed by the principal investigator and research analysts, and data extraction and analysis were completed using standardized procedures. All collected data were stored digitally in Excel spreadsheets in de-identified form, while personal identifiers were kept in a separate password-protected file accessible only to the principal investigator. Only the PI had access to the identifying personal information.

For each eligible patient, the date of surgery, the dates of the pre- and post-operative PHQ-9 assessments, and all item-level and total PHQ-9 scores were recorded. The dataset allowed for both aggregated comparisons of overall depressive symptoms and more focused analyses of individual questionnaire items. This structure ensured that changes in specific domains, such as somatic complaints, mood symptoms, or SDoH-related factors, could be evaluated alongside global PHQ-9 score changes following oral rehabilitation. For this specific analysis, average pre- and post-surgery PHQ9 scores were calculated and compared using Microsoft Excel (Version 2024; Microsoft Corporation, Redmond, USA) and Google Sheets (Google, Mountain View, USA).

## Results

A comparative analysis of pre- and post- surgery PHQ-9 scores was conducted to evaluate the impact of full-arch implant rehabilitation on depressive symptoms among individuals in prolonged recovery from substance use disorders. The modified PHQ-9, which incorporated items related to SDoH and subjective satisfaction with oral function, allowed for a broader assessment of patient experience before and after treatment. The data were organized according to whether the PHQ-9 scores improved, worsened, or stayed the same from pre- to post-surgery. The main goal of this analysis was to compare the objective outcome of the procedure (a medical success) to the subjective outcome of the procedure (patient satisfaction) by analyzing the observed differences between pre- and post-op PHQ-9 responses. 

All 101 patients included in the study reported ‘better’ scores post-op. Tables 1-3 provide an overview of the associated data, including average pre- and post- scores for each of the nine questions, a range of total scores, and average changes from pre- to post-op. Initial pre-surgery PHQ-9 scores for all patients ranged from 2 to 27, with 80% (n=81) exceeding the pre-established threshold of 20 or more. Post-surgery scores ranged from 0 to 8, with 92% (n=93) ranging from 0 to 4 (the lowest pre-established threshold). Average pre- and post-op scores were 24.16 and 0.75, respectively, indicating that the average patient improved their score by 23.41 points (out of 27 total, 86.7%).

**Table 1:** Group Level Survey Statistics Average pre and average post scores were calculated in Google Sheets and Microsoft Excel 2024 using the sum function on all pre and post scores, respectively.

Statistics	
Total Pre Surveys	N=101 (100%)
Total Post Surveys	N=101 (100%)
Total Full Cases (N)	N=101 (100%)
Average Pre Score	24.16
Average Post Score	0.75
Average Change	-23.41
Pre-Surgery Max Score	27
Pre-Surgery Min Score	2
Post-Surgery Max Score	8
Post-Surgery Min Score	0

**Table 2:** Average Score for Each Individual Question

Questions	Pre	Post
Q1	2.80	0.07
Q2	2.86	0.08
Q3	2.50	0.11
Q4	2.71	0.04
Q5	2.51	0.09
Q6	2.38	0.08
Q7	2.96	0.07
Q8	2.80	0.09
Q9	2.62	0.13

**Table 3:** Surveys Sorted by Score Ranges

Scores	Pre-surgery	Post-surgery
0-4	1	93
5-9	0	8
10-14	8	0
15-19	11	0
20-27	81	0

Finally, the analytic strategy was designed to contextualize the magnitude of pre- to post-op change within a broader landscape of PHQ-9 research. Prior studies of full-arch implant rehabilitation have consistently shown meaningful but variable improvements in patient-reported outcomes, with a proportion of individuals experiencing minimal or no change in satisfaction despite objectively successful surgery. By contrast, the present study population of individuals in sustained recovery from substance use disorders demonstrated uniformly completed assessments and exceptionally high engagement throughout the surgical and follow-up process. Further discussion is warranted to nail down the explanation behind this trend, but perhaps the motivation to rebuild one’s oral health during recovery reflects a distinct psychosocial profile, one that may amplify the perceived benefits of treatment and reinforce long-term sobriety.

## Discussion

SUDs severely compromise oral health through intertwined biological, behavioral, and social mechanisms. Chronic exposure to opioids, heroin, and alcohol disrupts normal salivary secretion, depresses immune function, and contributes to pronounced lapses in hygiene practices. Retrospective analyses demonstrate that patients in opioid addiction treatment programs exhibit substantially greater oral disease burdens, including median decayed-missing-filled-teeth scores more than double those of matched controls, increased plaque accumulation, reduced salivary pH, and elevated periodontal treatment needs [5]. Comparable patterns are observed among individuals with alcohol dependence, where malnutrition and microbial dysbiosis further intensify periodontal inflammation [17]. Periodontitis, one of the most prevalent non-communicable diseases globally, is strongly associated with systemic conditions such as diabetes, cardiovascular disease, and respiratory illness [18,19]. These oral-systemic interactions intersect with SDoH, as socioeconomic status, education, and access to care profoundly shape oral disease trajectories [20].

Determining when a recovering addict is an appropriate candidate for elective oral reconstruction depends largely on relapse risk trajectories. Relapse rates of 72-88 percent have been reported within three years following opioid detoxification when patients do not engage in medication-assisted treatment [21]. Similarly, more than 85 percent of abstinence episodes among heroin users have been shown to end in relapse within five years [22]. After approximately three years of sustained sobriety, however, relapse risk appears to decline, reflecting progressive consolidation of behavioral, psychological, and social stability. This three-year threshold underlies clinical programs such as Smiles for Recovery, which recommend implant rehabilitation only after prolonged, verifiable recovery. Individuals who surpass this interval are more likely to exhibit characteristics strongly predictive of long-term implant success: greater adherence to medical and dental follow-up, improved hygiene practices, and more consistent behavioral health patterns [21,22].

Oral inflammation serves as a potent systemic driver of disease through elevated circulating cytokines such as C-reactive protein (CRP), interleukin-1β (IL-1β), and tumor necrosis factor-alpha (TNF-α), which exacerbate insulin resistance and impair glycemic control [18,23]. Periodontal interventions in patients with type 2 diabetes have been shown to significantly reduce both glycated hemoglobin (HbA1c) levels and inflammatory markers [24]. The Renew Procedure, a full-arch anchored prosthesis, has shown similar outcomes, including clinically meaningful reductions in HbA1c following comprehensive removal of oral infection and rehabilitation [25]. Broad reviews corroborate this bidirectional relationship: diabetes accelerates periodontal destruction through hyperinflammatory pathways, while untreated periodontitis worsens metabolic regulation and increases the risk of poor glycemic control [26,27]. Restoring oral health produces systemic benefits extending well beyond improved mastication or aesthetics. 

Chronic systemic inflammation also contributes to depression, forming a biological link between oral disease, metabolic dysfunction, and mental health. Elevated levels of IL-1β, TNF-α, and CRP are consistently associated with greater depressive symptom severity and treatment resistance, indicating that a subset of depression is driven by inflammatory mechanisms [28]. A pooled analysis of 15 population-based cohort studies including more than 56,000 adults found that higher CRP concentrations were specifically linked to somatic depressive symptoms, such as changes in appetite, low energy, sleep problems, and loss of interest, suggesting a distinct inflammation-related depression phenotype [29]. Complementary meta-analytic evidence indicates that approximately 27% of individuals with depression exhibit low-grade systemic inflammation (CRP > 3 mg/L), a significantly higher prevalence than in non-depressed controls [30]. Importantly, reductions in inflammatory burden following oral rehabilitation may alleviate depressive symptoms. In a recent clinical study of the Renew Procedure, a full-arch implant-based rehabilitation, researchers observed significant improvements in PHQ-9 scores after treatment, suggesting that comprehensive oral restoration may function as both a physiological and psychosocial intervention for inflammation-linked depression [15].

Full-arch implant treatment reliably boosts how people feel about their mouths and their day-to-day lives. In one cohort of 99 patients, both the temporary and final implant bridges made it much less likely for individuals to report poor oral health-related quality of life [16]. Similar large improvements in PIDAQ-23 and OHIP-14 scores have been observed after Maxilla-for-All/All-on-X treatment [31], with the share of patients rating their oral condition as “unfavorable” dropping from 69 percent before surgery to 21.8 percent one year later, even when minor surgical or technical issues occurred. Other studies likewise document significant gains in eating comfort, confidence, and social engagement following implant placement, with patients reporting marked improvements in their ability to eat, smile, speak clearly, and interact socially without embarrassment [32]. Longitudinal analyses demonstrate persistence of these benefits: in a large survey 8 to 14 years after implant therapy, over 90% of patients remained satisfied or sufficiently satisfied with their implant restorations and reported high chewing comfort and enduring aesthetic satisfaction [33]. Notably, these effects extend to vulnerable groups, including edentulous individuals and those with complex medical or psychosocial histories, who often experience pronounced gains in psychosocial well-being, daily functioning, and self-sufficiency following implant-supported rehabilitation [34].

Oral health inequities mirror broader socioeconomic gaps. Fundamental social determinants, such as income, educational attainment, housing stability, food security, and access to transportation are significant predictors of whether U.S. adults obtain regular dental care, experience embarrassment regarding their oral health, and/or experience tooth loss [35]. Similarly, adults reporting poorer mental health were less likely to utilize dental services and more likely to have unmet oral health needs [36], underscoring the compounding effects of mental health challenges, poverty, and underutilization of care for oral disease. Vulnerable and marginalized populations, such as Syrian refugees and food-insecure older adults, show even stronger links between poor oral health and adverse mental health outcomes: poorer self-rated oral health is associated with higher levels of depression, anxiety, and stress among refugees, while food-insecure elders have significantly higher odds of gum disease and unmet dental needs [37,38]. These findings align with the SDoH framework articulated in Healthy People 2030, which emphasizes that equitable health outcomes require addressing upstream barriers such as economic stability, education, transportation, and access to care [20]. Integrating definitive dental rehabilitation into addiction recovery programs embodies this approach, offering measurable gains in physical function, psychological well-being, and socioeconomic stability while supporting community reintegration and long-term recovery.

The convergence of addiction, systemic inflammation, and oral disease represents an underrecognized but critical intersection in public health. Individuals recovering from SUDs often achieve psychological sobriety years before regaining full physiological stability, yet their oral health, which has often been devastated by years of neglect, remains a visible and functional reminder of addiction’s toll. The present findings show dramatic and near-universal reductions in depressive symptoms following full-arch implant rehabilitation among individuals in prolonged recovery and suggest that definitive oral reconstruction may serve as a powerful catalyst for both biological and psychosocial healing. An average PHQ9 decrease from 24 to less than 1 represents a shift from severe depression to minimal or no depressive symptoms for the vast majority of patients, far exceeding conventional thresholds for clinically meaningful change.

These outcomes are consistent with the broader mechanistic literature linking oral inflammation, metabolic dysfunction, and mood. Chronic periodontitis drives systemic elevations in inflammatory mediators such as CRP, IL-1β, and TNF-α, which worsen insulin resistance and glycemic control [18,23]. This, in turn, accelerates periodontal destruction through hyperinflammatory pathways, creating a vicious cycle in which oral disease and metabolic dysregulation perpetuate one another [26,27]. Parallel evidence shows that the same inflammatory mediators are tightly associated with depressive symptom severity and treatment resistance, and that a substantial subset of individuals with depression exhibit low-grade systemic inflammation [28-30]. Against this backdrop, the marked reduction in depressive symptoms observed after the Renew Procedure can be understood not as a purely cosmetic effect, but as the downstream result of removing a major chronic inflammatory focus, improving nutrition and glycemic control, and restoring social functioning [15,25].

The magnitude and consistency of PHQ-9 improvement in this cohort also align with prior work on full-arch implant rehabilitation in the general population. Large prospective studies have documented that implant-supported prostheses sharply reduce the odds of poor oral health-related quality of life and produce large gains in eating comfort, aesthetics, and social confidence [16,31,32]. Long-term follow-up surveys 8-14 years after implant therapy show that more than 90 percent of patients remain satisfied or sufficiently satisfied, with sustained chewing comfort and self-image benefits [33]. The current study extends these observations into a high-risk, high-need population (individuals in sustained recovery from SUDs) and suggests that when oral rehabilitation is timed to coincide with a period of behavioral stability (approximately three years of sobriety), the psychosocial and mental health gains may be especially pronounced. High engagement with follow-up visits and complete questionnaire capture in this cohort further indicate that patients who choose to pursue major oral reconstruction during recovery may represent a particularly motivated subgroup, one for whom treatment becomes a concrete symbol of identity change and long-term commitment to sobriety.

From a health equity perspective, these findings underscore how targeted oral rehabilitation can disrupt the cycle linking addiction, poverty, poor oral health, and mental illness. Social determinants such as income, education, housing stability, food security, and transportation access are closely tied to oral health outcomes and dental care utilization [35,36]. Vulnerable groups, including refugees and food-insecure older adults, show especially strong associations between poor oral health and depression, anxiety, and stress [37,38]. Individuals with SUDs frequently occupy the convergence of these disadvantages, facing barriers to employment, social integration, and routine care. By restoring dentition, mastication, and facial aesthetics, full-arch implant rehabilitation directly improves nutrition, communication, employability, and self-confidence, further contributing to sustained recovery and to closing long-standing gaps in oral and mental health outcomes.

From a policy standpoint, integrating definitive dental rehabilitation into addiction treatment and recovery programs aligns closely with the Healthy People 2030 social determinants of health framework, which emphasizes addressing upstream barriers (economic stability, education, transportation, and access to care) to achieve equitable outcomes [20]. The existing literature suggests that when major oral health problems are treated as part of a holistic approach to SUD care, patients remain in treatment longer and are more likely to complete recovery programs, with downstream reductions in health-care utilization and social service dependency. The present findings add to this evidence base by quantifying the mental health impact of full-arch rehabilitation in a real-world recovery population. Future work should compare different models of integrating implant rehabilitation into addiction care, rigorously evaluate cost-effectiveness, and explore how these interventions can be scaled in a way that preserves careful patient selection and high-quality surgical and prosthetic outcomes.

## Conclusions

Full-arch implant rehabilitation in people with sustained recovery from SUDs appears to do far more than restore teeth. In this cohort, comprehensive oral reconstruction was associated with clinically meaningful reductions in depressive symptoms, alongside large gains in function, aesthetics, and self-confidence. These changes are biologically plausible given the well-documented links between chronic periodontal inflammation, metabolic dysregulation, and inflammation-related depression, and they extend prior implant research by demonstrating particularly strong benefits when treatment is offered after approximately three years of continuous sobriety. At that point, relapse risk is lower, adherence to follow-up is higher, and patients are better positioned to maintain the hygiene and behavioral routines required for long-term implant success. Framed within an SDoH agenda, integrating definitive dental rehabilitation into addiction recovery programs offers a concrete way to reduce inflammatory burden, improve nutrition and employability, and narrow long-standing gaps in oral and mental health. For clinicians, health systems, and investors alike, these findings support viewing full-arch implant rehabilitation in stabilized recovery populations not as a luxury, but as a high-value, scalable intervention that can help convert years of addiction-related damage into durable biopsychosocial recovery.
